# Bipolar Membranes for Direct Borohydride Fuel Cells—A Review

**DOI:** 10.3390/membranes13080730

**Published:** 2023-08-13

**Authors:** Ines Belhaj, Mónica Faria, Biljana Šljukić, Vitor Geraldes, Diogo M. F. Santos

**Affiliations:** Center of Physics and Engineering of Advanced Materials, Laboratory for Physics of Materials and Emerging Technologies, Chemical Engineering Department, Instituto Superior Técnico, Universidade de Lisboa, 1049-001 Lisbon, Portugal; ines.belhaj@tecnico.ulisboa.pt (I.B.); monica.faria@tecnico.ulisboa.pt (M.F.); biljana.paunkovic@tecnico.ulisboa.pt (B.Š.); vitor.geraldes@tecnico.ulisboa.pt (V.G.)

**Keywords:** direct liquid fuel cells (DLFCs), direct borohydride–hydrogen peroxide fuel cells (DBPFCs), ion-selective membranes, bipolar membrane, pH-gradient, crossover, cation-exchange membrane, anion-exchange membrane

## Abstract

Direct liquid fuel cells (DLFCs) operate directly on liquid fuel instead of hydrogen, as in proton-exchange membrane fuel cells. DLFCs have the advantages of higher energy densities and fewer issues with the transportation and storage of their fuels compared with compressed hydrogen and are adapted to mobile applications. Among DLFCs, the direct borohydride–hydrogen peroxide fuel cell (DBPFC) is one of the most promising liquid fuel cell technologies. DBPFCs are fed sodium borohydride (NaBH_4_) as the fuel and hydrogen peroxide (H_2_O_2_) as the oxidant. Introducing H_2_O_2_ as the oxidant brings further advantages to DBPFC regarding higher theoretical cell voltage (3.01 V) than typical direct borohydride fuel cells operating on oxygen (1.64 V). The present review examines different membrane types for use in borohydride fuel cells, particularly emphasizing the importance of using bipolar membranes (BPMs). The combination of a cation-exchange membrane (CEM) and anion-exchange membrane (AEM) in the structure of BPMs makes them ideal for DBPFCs. BPMs maintain the required pH gradient between the alkaline NaBH_4_ anolyte and the acidic H_2_O_2_ catholyte, efficiently preventing the crossover of the involved species. This review highlights the vast potential application of BPMs and the need for ongoing research and development in DBPFCs. This will allow for fully realizing the significance of BPMs and their potential application, as there is still not enough published research in the field.

## 1. Introduction

The quest for sustainable energy sources that are efficient, renewable, and environmentally benign is now more imperative than ever. The massive use of fossil fuels leads to enormous carbon emissions, which have resulted in climate change (e.g., greenhouse effect and sea-level rise) [1]. Therefore, the concerns over energy sustainability and environmental issues are gaining increasing importance. Power generation from renewable and clean energy resources will definitely become the main energy source of future energy strategy [2]. Fuel cells have emerged as a viable alternative to conventional energy sources. The genesis of fuel cells traces back to 1839 when William Robert Grove discovered these electrochemical energy conversion devices. Traditional fuel cells primarily utilize hydrogen as their fuel source. Still, the challenges related to the storage and supply of hydrogen, primarily due to safety and efficiency concerns, have led to the exploration of other alternatives [3,4]. Among the diverse options, sodium borohydride (NaBH_4_) and methanol have shown promise as liquid fuels for direct liquid fuel cells (DLFCs), operating directly on liquid fuel instead of hydrogen, as in proton-exchange membrane fuel cells (PEMFCs). DLFCs have the advantages of higher energy densities and fewer issues with the transportation and storage of their fuels compared with compressed hydrogen and are adapted to mobile applications.

Specifically, direct borohydride fuel cells (DBFCs) employ (NaBH_4_) as a fuel and have distinct advantages in terms of energy density. The success of DBFCs, like other fuel cells, heavily relies on the electrochemical processes within the cell. A critical component that influences these processes is the separator or membrane used in the fuel cell. Initially, cation-exchange membranes (CEMs) were presumed to be more effective, particularly in controlling the crossover of negatively charged borohydride ions (BH_4_^−^), as opposed to anion-exchange membranes (AEMs) [5]. Nafion, a proton-exchange membrane (PEM), a subset of CEM, gained prominence as an effective separator for direct borohydride–hydrogen peroxide fuel cells (DBPFCs). However, as the research landscape evolves, Nafion faces competition from alternative membrane materials. This reflects a growing dynamism and diversity in membrane technologies that aim to address the challenges and limitations of conventional materials. The choice of the membrane is paramount, as it affects not only the efficiency but also the environmental impact and economic feasibility of the fuel cell applications [6,7,8].

CEM and AEM have served pivotal roles in the progression of DBPFCs, yet their inherent limitations necessitate a more refined approach. Bipolar membranes (BPMs) have appeared as an advanced combination of CEMs and AEMs. BPMs bring unique features to the maintenance of the critical pH gradient between alkaline BH_4_^−^ anolytes and acidic hydrogen peroxide (H_2_O_2_) catholytes, a requirement paramount to the effectiveness of electrochemical reactions at the electrodes. This ability is not only an incremental advance but also a fundamental enhancement that has the potential to redefine fuel cell performance and efficiency standards. 

The adoption of BPMs is not without challenges, and current research endeavors aim to optimize the balance between stability and selectivity in BPMs. These challenges are multifaceted and encompass various aspects. Firstly, manufacturing complexity presents a significant hurdle, as producing high-quality BPMs demands precise fabrication techniques and specialized materials, resulting in elevated production costs. Secondly, maintaining membrane stability under diverse operating conditions, such as high temperatures, corrosive environments, and exposure to reactive species, becomes paramount in ensuring long-term effectiveness. Achieving the desired selectivity for proton and hydroxide ion transport while minimizing the crossover of other species is an intricate task that affects the efficiency of BPMs in facilitating ion transport and separation. Furthermore, operating at high current densities (>1.5 A cm^−^^2^) poses a crucial challenge, requiring stable ion exchange performance for optimal fuel cell and electrolysis system functionality. Table 1 compares the performance of several DBPFCs reported in the literature, where the advantages of applying BPMs over CEM and AEM are clear.

With this, the stage is set for BPMs to potentially revolutionize electrochemical energy conversion and storage applications. This manuscript provides an in-depth analysis of the challenges and possibilities that BPMs bring to the forefront.

## 2. Processes Occurring in the Direct Borohydride–Hydrogen Peroxide Fuel Cell

Renewable energy sources play a critical role in addressing environmental challenges associated with conventional energy production by offering a sustainable and cleaner alternative. Unlike fossil fuels, which contribute to greenhouse gas emissions and climate change, renewable energy provides a greener option. However, the widespread adoption of hydrogen fuel cells, which offer renewable and green energy, faces obstacles such as cost and safety with its transport requirements. A potential solution lies in DLFCs utilizing methanol or ethanol, simplifying fuel storage for portable devices. DBPFCs, as low-temperature DLFCs utilizing NaBH_4_ and H_2_O_2_, offer advantages in terms of lower cost and lower emissions compared with ethanol and methanol fuel cells. However, they do not surpass hydrogen fuel cells in terms of energy density, with H_2_/air fuel cells able to generate 1 W cm^−^^2^ without harmful emissions. Despite slightly lower energy density, DBPFCs are still well-suited for space and underwater applications due to their cost-effectiveness, simplified fuel/oxidant management, and reduced emissions among DLFCs [8]. In a DBPFC, the borohydride oxidation reaction (BOR) at the anode is given by Equation (1):

BH_4_^−^ + 8OH^−^ → BO_2_^−^ + 6H_2_O + 8e^−^    E^0^ = −1.24 V vs. SHE (1)

where E^0^ represents the standard electrode potential of BOR against the standard hydrogen electrode (SHE). As there is always some production of hydrogen gas due to the unwanted partial hydrolysis of BH_4_^−^ (Equation (2)), the possibility of this reaction occurring as a parasitic reaction cannot be ignored. Thus, the borohydride oxidation considering the parasitic BH_4_^−^ hydrolysis can be written as Equation (3):

BH_4_^−^ + H_2_O → H_2_ + BH_3_OH^−^ (2)

BH_4_^−^ + xH_2_O → B(OH)_4_^−^ + (x − 4) H_2_O + (4 − x/2) H_2_ + xe^−^ (3)

where “x” is the coulombic number. The x value can be obtained experimentally using typical electrochemical methods, like cyclic voltammetry, rotating disk electrode (RDE), and rotating ring-disk electrode (RRDE) measurements, or by measuring the volume of hydrogen gas produced [15]; x values can range between 0 and 8, usually being 4 when using platinum-based electrodes [16].

Gold-based electrocatalysts are usually utilized to prevent BH_4_^−^ hydrolysis and were long considered the most faradaic efficient. In alkaline media, H_2_O_2_ exists as HO_2_^−^ (Equation (4)), which can be reduced via a two-electron process to hydroxyl ions, as shown in Equation (5). The cathodic reaction in a DBPFC is influenced by the pH conditions described in Equations (5) and (6) for alkaline and acidic media, respectively. At this high pH, AEMs are more adequate than CEM because the OH^−^ ions generated during the hydrogen peroxide reduction reaction (HPRR) pass through the AEM and are consumed in the BH_4_^−^ oxidation reaction.

H_2_O_2_ + OH^−^ → HO_2_^−^ + H_2_O (4)

HO_2_^−^ + H_2_O + 2e^−^ → 3OH^−^    E^0^ = 0.87 V vs. SHE (5)

Although this seems like an elegant concept, the low stability of H_2_O_2_ in alkaline media leads to its spontaneous decomposition. Thus, the system’s efficiency must be increased by keeping the oxidant solution at low pH. However, under acidic conditions, H_2_O_2_ is adsorbed onto the electrode and is reduced according to Equation (6).

H_2_O_2_ + 2H^+^ + 2e^−^ → 2H_2_O E^0^ = 1.77 V vs. SHE (6)

Combining Equations (1) and (6) leads to the net cell reaction of a typical alkaline–acidic DBPFC, as given by (Equation (7)), with a theoretical cell voltage of 3.01 V at 25 °C [8].

BH_4_^−^ + 4H_2_O_2_ → BO_2_^−^ + 6H_2_O E_0_ = 3.01 V (7)

Šljukić and Santos have argued that the preferred path for H_2_O_2_ electroreduction in fuel should be in acidic media (Equation (6)), involving the direct pathway and faster kinetics [8]. Additionally, the direct pathway yields a higher cell voltage due to the higher standard electrode potential of the HPRR. However, the indirect mechanism, which occurs in alkaline media, presents added requirements and greater complexity in constructing the fuel cell system, primarily due to gas management considerations. All three mass-transfer processes occur within a DBPFC’s electrolyte: (electro)migration, convection, and diffusion. In the case of a CEM, the consumption of OH^−^ in the BOR leads to a decrease in the number of anions on the anode side. 

**Figure 1:**
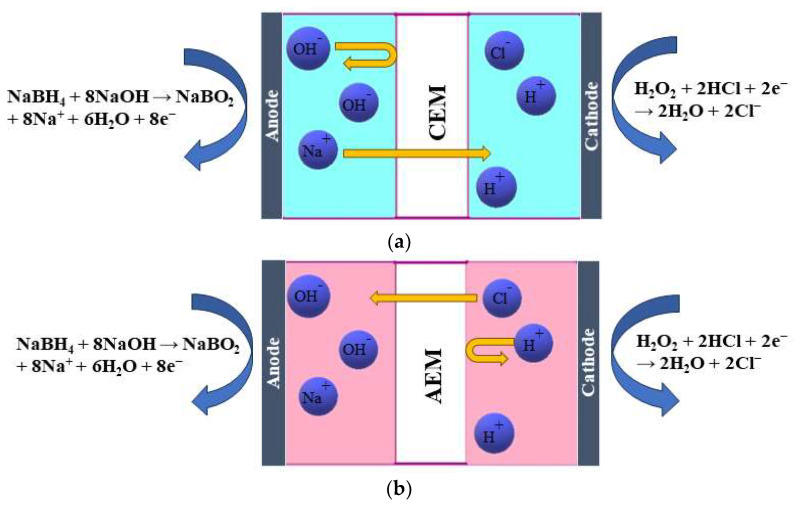
Schematic illustration of the working principle of (**a**) a cation-exchange membrane (CEM) and (**b**) an anion-exchange membrane (AEM) in a DBPFC.

Cations must be sent away to compensate for this, resulting in Na^+^ ions crossing the membrane from the anode to the cathode compartment (Figure 1a). This process is based on diffusion. Additionally, a concentration gradient of other species, such as BH_4_^−^, is present, and by diffusion, BH_4_^−^ ions tend to move to the other side. However, the presence of a CEM only allows the passage of cations, not anions, thus preventing the BH_4_^−^ crossover to the other side. The presence of an AEM allows only the passage of OH^−^ and Cl^−^ [15] (Figure 1b). Those are based on the Donnan exclusion principle, which states that only ions with opposite charges can be transferred, while transferring ions with the same charge as the membrane-immobilized group is largely prohibited. Therefore, the migration of ions across a membrane is caused by the electric field that arises from the anodic and cathodic reactions. The type of membrane separator used determines the charge carrier. As discussed previously, in the case of an AEM, OH^−^ and Cl^−^ anions move from the cathodic to the anodic compartment to maintain the cell’s charge balance. At the same time, Na^+^ cations cross the CEM in the cathodic direction to achieve the same result. Additionally, neutral species such as H_2_O_2_, gases, and organic compounds may diffuse from one compartment to the other due to concentration gradients [17].

## 3. Trends of Membranes as Separators in DBPFCs

The membrane separator is a crucial component in DBPFCs as it prevents shorting between the anode and cathode and the mixing of fuel and oxidant solutions. The ion-selective membranes used in fuel cells can be classified into CEMs, AEMs, and BPMs. Each type of membrane has its own specific characteristics and applications. Alkaline–acidic DBPFCs use NaBH_4_ in sodium hydroxide (NaOH) solution as the alkaline fuel and H_2_O_2_ in hydrochloric acid (HCl) as the oxidant. The processes occurring inside the cell will differ based on the ion-selective membrane used. An AEM selectively allows the passage of negatively charged ions (anions), specifically, in this DBPFC, OH^−^ and Cl^−^ anions can move through the AEM to the anode. The anions movement helps to maintain the charge balance within the fuel cell. On the other hand, using a CEM will selectively allow the passage of positively charged ions (cations). In such a DBPFC, Na^+^ cations move from the anode to the cathode through the CEM. Neutral species, such as H_2_O, gases, or organic compounds, diffuse across the membrane due to concentration differences between anodic and cathodic compartments [15]. So, by separating the anode and cathode compartments and controlling the ion flow, the membrane separator enables the efficient operation of the DBPFC. It allows the necessary ion transport while preventing unwanted reactions and ensuring the overall performance of the fuel cell. The most widely used membrane in DBFCs and fuel cells, in general, is based on Nafion, a perfluorinated compound, due to its good ionic conductivity, enhanced chemical and thermal stability, and low fuel permeability. For example, the BH_4_^−^ permeability in the Nafion^®^117 membrane is 8.8 × 10^−^^9^ mol cm^−^^1^ s^−^^1^ [18]. This allows DBPFCs using Nafion membranes to achieve peak power densities of up to 390 mW cm^−^^2^ at 80 °C [10]. However, a significant issue with using CEMs in DBPFCs is that it decreases the alkali concentration in the anolyte, resulting in instability and ineffective use of NaBH_4_ as fuel. The oxidation of 1 mole of NaBH_4_ requires the transfer of 8 moles of Na^+^ across the membrane, resulting in an increase in NaOH in the catholyte and a decrease in the anolyte [19]. This problem becomes increasingly severe with longer operation times [20].

Nafion^®^961 membrane has been used instead of Nafion^®^117 to reduce alkali crossover from anodic to cathodic compartments of the cell [19]. Nafion^®^961 is a Teflon-fiber-reinforced composite membrane with sulfonate and carboxylate polymer layers. The carboxylate layer of Nafion^®^961 resists the flow of NaOH from the anode to the cathode, thereby improving the cathode polarization behavior of the DBPFC. Chemours Nafion^®^1100 EW series is also considered a suitable separator, but it leads to chemical imbalances [19]. To maintain stability during an extended fuel cell operation, there must be a way to return NaOH from the catholyte to the anolyte, which is difficult to achieve. AEMs may be used to maintain the chemical balance, but they often lack stability in alkaline conditions; for instance, existing AEMs in the market struggle to endure hydroxide concentrations beyond approximately 5%. For example, the A-501SB perfluorinated anion membrane developed by Tosohfi served as an effective separator, but its production costs halted its usage [19]. In this context, researchers have attempted to employ various optimized techniques by conducting multiple experiments and adjusting the parameters to enhance the model’s performance. For instance, to enhance the proton conductivity in PEM fuel cells, doped Nafion membranes were used; the dopants were sourced from aryl mono or bis phosphonic acid and incorporated into the membranes through impregnation or casting techniques. The proton conductivity was evaluated using electrochemical impedance spectroscopy, allowing for an assessment of the effect of structure and preparation method on proton transport. The results indicated that the proton conductivity of the membranes produced through casting was higher than commercially available Nafion^®^N-115 from Chemours [21]. In a different study, Teixeira et al. prepared different indazole and benzotriazole bis phosphonic acids for incorporation into Nafion^®^N-115 membranes, with a maximum loading of 5%. The Nafion-azole bisphosphonate membranes with 1% loading showed improved proton conductivity compared with the reference Nafion^®^N-115. The membrane containing [hydroxy(1H-indazol-3-yl) methanediyl] bis (phosphonic acid) displayed a proton conductivity of 98 mS cm^−^^1^ [22]. However, composite membranes using crosslinking and casting methods were prepared in another study; they synthesized a ternary crosslinked polymer from inexpensive and readily available polymers, including polyvinyl alcohol (PVA), polyethylene oxide (PEO), and polyvinyl pyrrolidone, and added sulfonated graphene oxide (SGO) as a dopant to the polymer matrix. Incorporating SGO in the membrane reduced the swelling ratio to 17% and decreased BH_4_^−^ permeability. The peak power density of a DBPFC with such a membrane was 65 mW cm^−^^2^, which was close to that of a DBPFC with Nafion^®^117 (81 mW cm^−^^2^) under the same testing conditions [23]. In a different study, research was conducted using (PO_4_–TiO_2_) and (SO_4_–TiO_2_) nanotubes as dopants to a PVA-based ternary blend used in their previous work. DBFCs operating with these membranes achieved power densities similar to those recorded with a Nafion^®^117 membrane but at a significantly lower cost [24].

Conversely, a binary polymer blend was created from low-cost and environmentally friendly polymers, PEO and PVA, and doped with phosphate titanium oxide nanotubes (PO_4_TiO_2_) at a concentration of 1–3 wt.%. The power density of a DBPFC with the PVA/PEO/PO_4_TiO_2_ blend was 72 mW cm^−2^, comparable with that of Nafion^®^117 (91 mW cm^−2^) under the same testing conditions [9]. These results paved the way for sustainable, economic, and straightforward approaches to creating composite membranes for practical DBPFCs. Though crosslinking polymer blends used to produce stable membranes had competed with the commercial Nafion, thin films were formed on a glass substrate by mixing the polyether (SFS) and poly(4,4′-diphenylether-5,5′-bibenzimidazole) (OPBI) in dimethylsulfoxide (DMSO); subsequent sulfuric acid post-treatment led to an ionically crosslinked membrane. In contrast, the covalent crosslinking of another membrane was achieved by adding 3,6-dioxa-1,8-octanedithiol to an SFS solution in DMSO, followed by a 1,8-diazabicyclo[5.4.0]undec-7-ene (DBU) solution in DMSO. The synthesized membranes outperform the commercial Nafion by increasing the maximum power density of a cell by up to 10%, resisting dissolution stress up to 84 wt.%, and reducing fuel crossover by up to 75–100% [25].

Conversely, an ion-pair-coordinated membrane (IPM) was developed by crosslinking OPBI with poly (vinylbenzyl chloride) (PVBC) and quaternising it with three amines (DABCO, quinuclidine, and quinuclidinol); this IPM system showed reduced swelling, improved mechanical properties, and better fuel cell performance than a commercial PBI membrane (i.e., Dapazol) [26].

The durability of membranes is an important matter in DBFCs and critical for their commercial success. Further studies on their stability over long-term operation are necessary, as performance degradation over time is common in fuel cells, including DBPFCs. It is often due to catalyst deactivation, changes in electrode porosity, and electrolyte degradation. Choosing the right membrane is vital to avoid such issues. Operating with Na^+^ or K^+^ electrolytes and an MOH (M = Na^+^ or K^+^) concentration of 10–40 wt.% and MBH_4_ concentration of 10–30 wt.% is generally recommended [22]. For maximum efficiency, a fuel cell membrane must have high ionic conductivity for high current flow with minimal resistive losses and no electronic conductivity, as well as strong mechanical stability, high oxidant permeability, high Coulombic efficiency, and low internal friction.

For maximum efficiency, a fuel cell membrane must have high ionic conductivity for high current flow with minimal resistive losses and no electronic conductivity, as well as strong mechanical stability. However, it should be clarified that the oxidant permeability needs to be controlled and balanced carefully. Too high permeability may lead to undesirable crossover reactions, where the oxidant leaks from the cathode to the anode, reducing fuel cell efficiency. On the other hand, too low permeability may hinder the efficient supply of oxidant to the electrode, affecting overall performance [20].

It is known that membrane thickness affects fuel cell performance; however, this is a complex issue, as thicker membranes reduce fuel/oxidant crossover but increase electrolyte resistance [7]. Moreover, high NaBH_4_ and H_2_O_2_ concentrations lead to higher cell voltages but also promote reactant crossover.

## 4. Why Bipolar Membranes in DBPFCs?

Generally, NaBH_4_ has two potential applications as a fuel for fuel cells. It can be used as a hydrogen source, through its hydrolysis reaction, for feeding conventional fuel cells. Secondly, it can be directly oxidized in an alkaline solution in the DBFC, as described in the previous section. The latter approach offers a higher theoretical cell voltage and lower system complexity. The fuel solution must be stabilized with an alkaline supporting electrolyte to prevent decomposition into B(OH)_4_^−^ and hydrogen. However, some hydrogen evolution occurs even during operation in highly alkaline media. The reduction of H_2_O_2_ can be catalyzed in both acidic and alkaline media, each offering different reduction potentials. As mentioned above, alkali media is preferred for sustainable pH balance and optimal stoichiometry, but it leads to cathode instability, oxygen evolution, and heat production. Acidic media provides advantages in oxidant stability and catalysis. Still, the requirement of adding NaOH for the anode reaction and acid for the cathode reaction adds weight and harms energy storage. To overcome these challenges, the use of BPMs in DBPFCs is proposed. The BPM enables alkaline fuel oxidation and acidic oxidant reduction without the need for storing large amounts of acid and alkali as supporting electrolytes. The BPM achieves this by splitting water into protons and hydroxide ions when a current passes through it [27]. The primary purpose of a BPM is to facilitate disproportionation reactions, in which water is electro-dissociated into protons and hydroxide ions at the bipolar junction. DBPFCs have been demonstrated primarily using a CEM or an AEM, as both enable converting the fuel’s chemical energy into electricity. BPMs combine a CEM and an AEM and can provide unique benefits for fuel cell applications [27]. First, researchers reported that the conflicting pH requirements for the oxidation of BH_4_^−^ (highly alkaline) and reduction of H_2_O_2_ (highly acidic) are hindering the performance and efficiency of DBPFCs. To address this issue, those researchers created a pH-gradient-enabled microscale bipolar interface (PMBI), which allows for distinct local pH environments at the anode and cathode. The PMBI was made up of a commercial CEM and a thin AEM and produced a sharp local pH gradient that improved the performance of the DBPFC. The DBPFC with PMBI reached a peak power density of 630 mW cm^−^^2^ at 1.0 V. Though this study offered promising results in improving fuel cell performance through customizing BPM interfaces, some challenges still need to be addressed [28].

**Figure 2:**
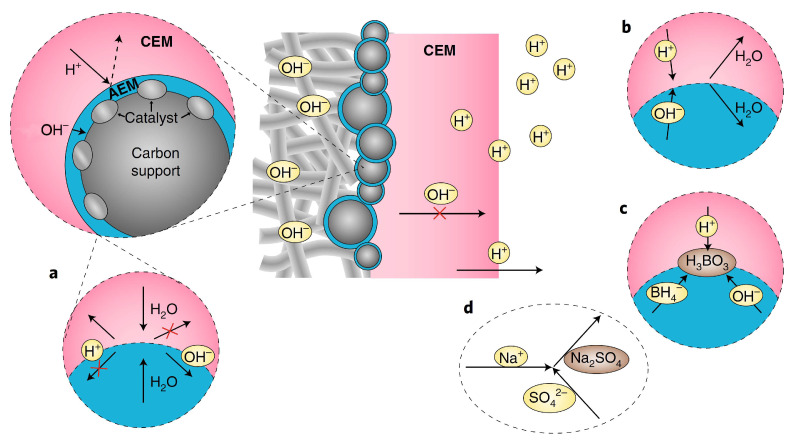
Representation of the different processes taking place at the pH-gradient-enabled microscale bipolar interface (PMBI) under the current flow. (**a**) Autoprotolysis of water occurs at the AEM|CEM interface, where H^+^ cannot be transported in the AEM and OH^−^ cannot be transported in the CEM (**b**,**c**). In open-circuit conditions at the AEM|CEM interface, diffusion of anions in the AEM and of cations in the CEM yield the consumption of acid of the catholyte and base from the anolyte into water (**b**) and formation of boric acid (H_3_BO_3_) from BH_4_^−^ reaction with OH^−^ and H^+^ that diffuse in the AEM and CEM, respectively (**c**,**d**). Details of the possible formation of Na_2_SO_4_ in the catholyte via the crossover of Na^+^ through the thin AEM, either under current or at open-circuit conditions. Adapted with permission from Ref. [15]. 2019, Springer Nature.

Firstly, the autoprotolysis (Figure 2a) of water and fast water transport is limited in PMBI, as H_2_O species must be transported to the CEM|AEM junction and decompose into H^+^ and OH^−^ to ensure current transport in the CEM and AEM. However, how to increase the autoprotolysis of water to reach high current densities is yet to be seen. Secondly, the study assumed perfect BPM permselectivity in ion transport, meaning only cations should migrate in the CEM and only anions should migrate in the AEM. Still, the anolyte and catholyte might diffuse into the “electrolyte drenched” BPM in actual DBPFCs, leading to consumption of the catholyte’s acid and the anolyte’s base, thus forming water in the junction region (Figure 2b). In the open-circuit situation, where there is a potential for ion diffusion due to the concentration gradient between the anolyte and the catholyte, this is more likely to be the situation. As a result, if the energy density of the DBPFC was to be determined, the computation should consider the entirety of the anolyte and catholyte, as well as the fuel and oxidant molecules, to enhance fuel/oxidant Faradaic efficiencies and to avoid parasitic processes such as BH_4_^−^ hydrolysis (Figure 2c). Additionally, Na^+^ can penetrate both the CEM and AEM sections, forming Na_2_SO_4_ as a parasitic product when it reacts with SO_4_^2−^ (Figure 2d). Thicker AEM may alleviate these problems but will affect the BPM resistance. The optimization of the electrocatalysts used at the two electrodes could further enhance the cell performance [12,29]. Despite the unavoidable junction potential of a BPM, the proposed PMBI design provides a new and exciting approach for creating fuel cell membrane electrode assemblies [15].

The reaction at the bipolar junction is referred to as both “water dissociation” and “water splitting” in the literature. However, the term “water dissociation” emphasizes that the reaction results in ion formation, not the evolution of H_2_ and O_2_ gas at the electrodes. Several mechanisms are believed to be responsible for generating H^+^ and OH^−^ ions. BPMs have a current–voltage curve similar to a p-n junction in a semiconductor diode. The chemical equilibrium equations of charge carriers in semiconductors are similar to those describing proton and hydroxide ion equilibrium in water, leading to the analogy of a BPM as a semiconductor p-n junction. The rate of water dissociation depends on the electric field in the bipolar junction. The Wien effect is a mechanism used to describe the water dissociation in the BPM interface, which is a forced dissociation of water molecules by an electric field [30]. The protonation–deprotonation mechanism suggests that H^+^ and OH^−^ ions can be produced in proton transfer reactions between water and fixed charged groups, with the involvement of a catalyst in water dissociation. This theory is supported by studies on the catalytic activity of various ionic groups, the observed larger water dissociation effect for one type of charged group, and improved water dissociation after adding a catalyst. Z. Yan et al. highlighted the significance of catalyst particles in accelerating water dissociation at the hybrid BPM junction. The presence of a catalyst layer, particularly graphene oxide (GO), reduces the electric field at the interface, improving BPM performance. BPMs with a catalyst layer exhibit a lower potential drop and higher stability than those without the added catalyst [31]. Fixed charges in the AEM and PEM create an electrochemical effect at the junction between the membranes. This behavior at the interface is comparable with that of a p-n junction in a semiconductor, as described previously, where H^+^ ions are the positive carriers in the PEM and OH^−^ ions are the negative carriers in the AEM. At the junction between an ideal PEM and AEM, the mobile protons in the PEM and mobile hydroxide in the AEM can combine to form water.

**Figure 3:**
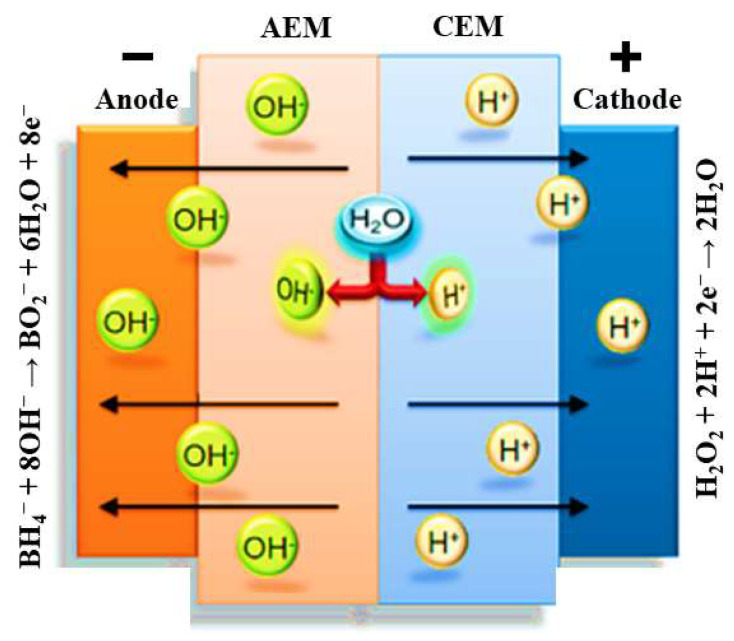
Schematic representation of the processes occurring at the electrodes and membrane of a DBPFC using a bipolar membrane.

The immobile sulfonate groups in the PEM, which have a negative charge, generate an electric field that counteracts the diffusion of more protons from the PEM to the PEM/AEM interface [32]. Consequently, the current flow in the fuel cell relies on the continuous generation of protons and hydroxide from water dissociation at the AEM/PEM interface (Figure 3). Thus, the steady-state operation of the cell is limited by the flux of water to the AEM/PEM interface [32].

It has been reported that DBPFCs recently achieved the highest power density of 630 mW cm^−^^2^ using a BPM [29]. However, the need for BPMs to be capable of handling further higher current densities should be recognized. Future research needs to be aware of the limitations of commercial membranes and to enhance the membrane performance to operate at such high power densities along with improving the membrane’s durability.

## 5. Synthesis of Bipolar Membranes

**Figure 4:**
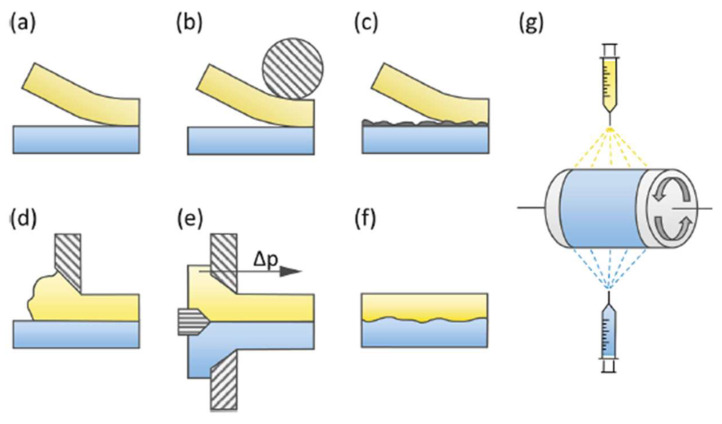
Bipolar membrane preparation methods: (**a**) loose lamination, (**b**) (hot) pressing, (**c**) lamination with a binder, (**d**) casting, (**e**) co-extruding, (**f**) modifying the opposite sides of a basis membrane, and (**g**) electrospinning. Reprinted with permission from Ref. [30]. 2021, Elsevier.

Various techniques can be used to produce BPMs, as shown in Figure 4. These techniques include hot or cold pressing/lamination (Figure 4a,b). For example, Nafion/FT-FAA and Nafion/FT-FAS BPMs were prepared by combining two commercially available ion-exchange membranes: the FT-FAA or FT-FAS anion-exchange membrane obtained from FuMA-Tech, and the Nafion^®^115 or Nafion^®^117 CEM acquired from Chemours [30]. To connect the AEM and CEM, a hot-pressing technique was employed. Before hot-pressing, the AEM was rinsed in 1 M NaOH solution, while the CEM was rinsed in a 1 M HCl solution. This rinsing process substituted undesirable ions within the membranes with OH^−^ and H^+^ ions [31]. Respectively, in the same context, BPMs were made from Nafion^®^NR-211 and hexamethyl-p-terphenyl poly(benzimidazolium), referred to as PBI. The PBI was converted to the OH^−^ form by soaking the membrane in 1 M potassium hydroxide (KOH) for 48 h at room temperature, followed by rinsing several times with water. Nafion membranes were converted to the H^+^ form by soaking in 1 M sulfuric acid (H_2_SO_4_) for 24 h and then rinsing several times with water. After rinsing, the membrane was soaked in water before use. The BPMs were prepared by manually laminating PBI and Nafion membranes without using any binder or applying pressure or heat [32]. Lamination with a binder can also be an efficient technique (Figure 4c) [33]; for instance, BPMs were made using Neosepta AHA membranes (anion-exchange layer, AEL) and Nafion^®^NR-211 (cation-exchange layer, CEL) to form AEL−GO, where a solution of graphene oxide (GO) was spin-coated onto the surface of the membrane. A Nafion contact layer was applied by spin-coating two layers of Nafion aqueous dispersion onto the AEL−GO surface. The CEL was attached by adding 0.5 mL of Nafion dispersion to the top layer of spin-coated Nafion and spreading a Nafion^®^NR-211 sheet [34]. The casting method (Figure 4d) has been used to fabricate cost-effective BPMs. In the first step, anion-exchange resin powder was added to the polymer solution, and the mixture was maintained at 60 °C for 5 h. Subsequently, the resulting mixture was applied onto a commercial CEM and left to dry completely at room temperature for 24 h. Similarly, the second type of BPM was produced by dispersing the cation-exchange resin powder in the polystyrene solution, which was prepared using the ethylene dichloride and toluene mixture. This mixture was then cast onto a commercial AEM [35]. It has been demonstrated that sulfonated poly(ether ether ketone) (SPEEK) presents several key advantages over Nafion membranes, including cost-effectiveness, attributed to the aromatic backbone, acceptable proton conductivity, higher selectivity, resulting from different nanophase separation, and notable mechanical and chemical stability, making it a promising candidate for fuel cells [36] and other electrochemical energy conversion applications [37]. Co-extruding (Figure 4e) and modifying a monopolar membrane using chemical or radiation-induced grafting (Figure 4f) can also be a way to synthesize BPMs. On the other hand, employing the electrospinning technique (Figure 4g) to fabricate BPMs can also be an efficient approach; for example, BPMs were synthesized using single- and dual-fiber electrospinning methods followed by subjecting the membrane to dimethylformamide vapor and hot-pressing. SPEEK was utilized as the cation-exchange polymer, while quaternized poly (phenylene oxide) was employed as the anion-exchange material. Al(OH)_3_ nanoparticles were integrated into the junction to facilitate the process of water splitting. The resultant trilayer membrane showcased an internal bipolar junction consisting of interpenetrating nanofibers composed of anion-exchange and cation-exchange polymers [35].

Recently, multilayered membranes were prepared using the spin coating method, incorporating Kevlar nanofibers, CdTe nanocrystals, and phosphoric acid (PA). PA played a key role in enhancing proton conductivity, resulting in impressive values of 2.35 × 10^–1^ S cm^−^^1^ at 160 °C for (Kevlar-CdTe-PA_)4_/PA membranes. The layered distribution of components also improved mechanical strength, with a tensile stress value of 2.29 MPa at room temperature, and the vacuum-assisted flocculation was used to create multilayered PEMs with poly(vinyl alcohol), Kevlar nanofibers, and carbon nanotube oxides [38]. Introducing phosphoric acid improved proton conduction, resulting in high and stable conductivity at subzero temperatures, making them ideal for low-temperature PEMFCs [39]. However, single-layer BPMs are advantageous because they are typically thinner, allowing easier water diffusion into the bipolar junction; research was conducted to construct interfaces for BPM with a controllable thickness at the junction; and it was indicated that membranes comprising two polyelectrolyte interfacial bilayers exhibited higher efficiency than membranes with more bilayers. Interestingly, increasing the contact area by adding more bilayers did not reduce the voltage drop across the BPM. It was also demonstrated that as the thickness of the junction increased, the BPM efficiency decreased. Furthermore, it was noticed that an increase in the number of bilayers led to an enhanced limiting current density [27]. Consequently, employing the layer-by-layer casting technique shows promise for manufacturing BPMs with desired properties. In the same context, it was also observed that as the number of bilayers increased, there was an increase in the limiting current density [12]. This finding further supports the potential of layer-by-layer casting as a promising technique for fabricating BPMs with the desired properties. Incorporating these advantageous techniques into BPMs makes it possible to pave the way for developing sustainable DBPFCs.

## 6. Methods for Membranes Characterization

**Figure 5:**
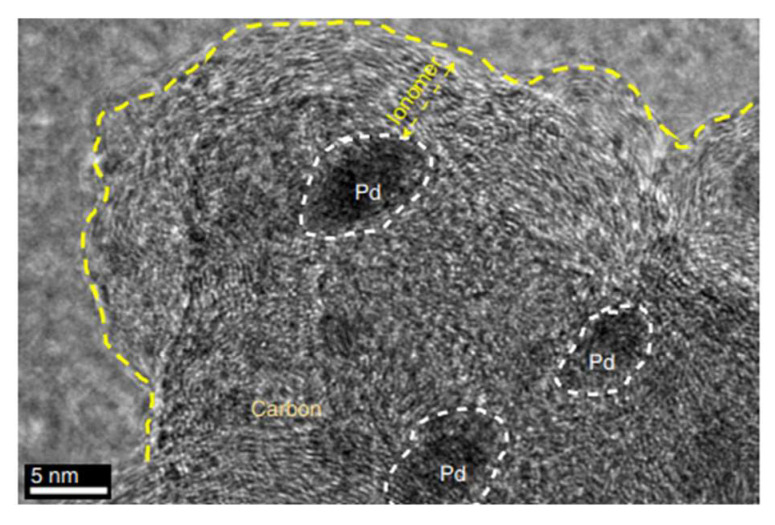
Transmission electron microscopy image of Pd/C covered by the anion exchange ionomer (polystyreneblock-poly(ethylene-ran-butylene)-block-polystyrene functionalized with tri-methylamine (SEBS55-TMA). Reprinted with permission from Ref. [29]. 2019, Springer Nature.

**Figure 6:**
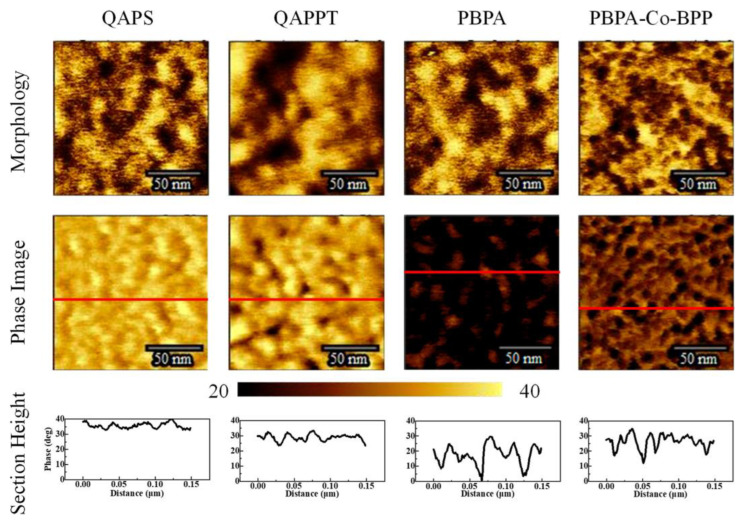
Atomic force microscopy analysis of morphology, phase, and section height of QAPS/QAPPT/PBPA/PBPA-Co-BPP membranes. Reprinted with permission from Ref. [45]. 2023, The American Chemical Society.

Investigating water dissociation and ion transfer in membranes can involve various techniques, including voltammetry, chronopotentiometry, electrochemical impedance spectroscopy (EIS), and methods for determining ion transport numbers. To fully understand processes in BPMs, a combination of these experimental methods is needed. EIS is a technique that measures the system’s response to alternating electric current at a given frequency. Currently, the focus of EIS in membrane systems is (1) measuring the resistance of ion-exchange membranes, (2) studying the transport of salt ions and water dissociation products in electro membrane systems, and (3) studying the rate constants of water dissociation and the structure of the bipolar region. The data obtained from EIS are often interpreted using equivalent circuits, requiring rigorous mathematical modeling [36,40,41,42]. To evaluate the ionic conductivity of membranes using EIS, samples have been pre-treated in 4 M NaOH solution and positioned between two stainless steel electrodes, using open circuit potential in the frequency range from 100 Hz to 100 kHz [23]. The resistance of the membranes was calculated from the high-frequency intercept on the real axis of the complex impedance plot, and the ionic conductivity was derived from membrane resistance, R, using Equation (8):

σ = d/RA(8)

where σ represents the ionic conductivity, A is the membrane area, and d is its thickness. In a different study [22], Nafion membranes were modified with phosphonic (PA) and bis phosphonic acid (BP) groups, and their proton conductivity was evaluated using EIS. The measurements were conducted at different temperatures (30 to 60 °C) and relative humidities (40 to 80%) in a climatic chamber. The proton conductivity was calculated using Equation (8). The equivalent circuit includes RC circuits representing the bulk and interfacial properties of the polymer, which was adjusted using a non-linear least square fitting technique provided via the ZView software. Chronopotentiometry is also used to study ion transport and chemical reactions in IEM and surrounding solution layers; it involves measuring the potential difference across the membrane over time under a constant current density. This method only applies to BPMs in neutral saline solutions, without causing damage [39,43,44]. Furthermore, the thermal stability of the membranes may be determined via a thermogravimetric analysis [38], their morphology can be analyzed via scanning electron microscopy [45], and their topography can be assessed via transmission electron microscopy (Figure 5 [29]) and atomic force microscopy (Figure 6 [45]).

Water uptake is the process of absorbing or taking in water. Understanding water uptake in membranes is important as it can lead to degradation and can affect the membrane properties. Water uptake can be determined using Equation (9):

% water uptake = ((W_w_ − W_d_)/W_d_) × 100 (9)

where W_d_ is the weight of the dried membrane sample and W_w_ is the weight of the wet membrane sample. For example, Gouda et al. reported water uptake values ranging between 15 and 100% [9].

The ion exchange capacity (IEC) is another important membrane property, indicating its capacity to exchange ions [23]. It could be estimated through acid–base titration, followed by the application of Equation (10):

IEC (meq g^−1^) = V_b_ × C_b_/W_d_ (10)

where V_b_ is the consumed base volume and C_b_ is the base concentration. The IEC values for PVA/PEO/PVP membranes prepared by Gouda et al. [9] ranged from 0.10 to 0.15 meq g^−1^, while Nafion^®^117 has a significantly higher IEC of 0.89 meq g^−1^.

The chemical stability of the membrane can be investigated by immersing the membrane in a Fenton reagent solution for 24 h and then comparing the dry weight of the membrane after exposure to the Fenton reagent with its initial dry weight (Equation (11)).

% oxidative stability = (W_d_ after Fenton test/initial W_d_) × 100 (11)

Yogarathinam et al. observed that the oxidative stability of the different modified SPEEK membranes gradually increased as modifications were made, with PANI-A-BN/SPEEK-2 showing the highest oxidative stability at 95% [36]. In fact, oxidative stability is an essential property for membranes to be employed in DBPFCs, due to the critical challenges arising from the presence of reactive oxygen species, such as peroxides and hydroperoxides. These species can cause membrane degradation and can diminish the cell’s performance over time. Oxidative stability is a crucial property that refers to the membrane’s ability to withstand and counteract the harmful effects of these reactive species, thereby preserving its structural integrity and functionality throughout the cell’s operational lifespan. Yogarathinam et al. recognized that significant progress in advancing the performance, reliability, and durability of DBPFCs can be achieved by studying and enhancing the oxidative stability of SPEEK membranes [36]. By improving oxidative stability, DBPFCs can benefit from extended cell lifetime, reduced maintenance costs, and overall enhancements in fuel cell efficiency. As a result, DBPFC will become more appealing for practical applications [36]. Finally, single-cell tests, including polarization curves and power density curves, must be studied to assess fuel cell performance using the developed membranes [29,46].

## 7. Conclusions

This review summarized various types of membranes examined for their suitability in DBPFCs and underscores the significance of using BPMs in such systems. A key advantage of employing BPMs in DBPFCs is their ability to maintain the necessary pH gradient between the alkaline BH_4_^−^ anolyte and the acidic H_2_O_2_ catholyte. This pH gradient is critical for facilitating electrochemical reactions at the anode and cathode. By effectively segregating the anode and cathode compartments, BPMs enable selective ion transport while preventing cross-contamination and unwanted side reactions. This feature leads to improved fuel cell performance and enhances overall efficiency. In recent years, the research interest in BPM has experienced rapid growth. The field of membrane synthesis and its applications are closely intertwined. Various synthesis techniques have enabled the widespread use of BPM beyond DBPFCs; these diverse applications have demanded the development of different types of BPM to meet specific requirements. Although green synthesis of BPM holds promise for the future, it is still in the early stage, and further research is needed to explore renewable membrane materials and to develop synthesis methods that are organic solvent-free.

To enhance the efficiency of DBPFCs, future research should focus on exploring cost-effective membrane materials with high ionic conductivity, high permselectivity, high stability, and long durability. In conclusion, this work underscores the importance of utilizing BPMs, particularly CEM/AEM-based BPMs, in DBPFCs to maintain the necessary pH gradient. This technology offers immense potential for advancing the field and revolutionizing energy conversion systems. By delving into future trends and applications of BPMs in DBPFCs, researchers can drive innovation, contribute to sustainability efforts, and propel the development of efficient energy solutions.

## Figures and Tables

**Table 1:** Membranes used in DBPFCs reported in the literature.

Membrane Designation	Type	Thickness/μm	Power Density/mW cm^−2^	T/°C	Anode Material	Cathode Material	Source
PVA/PEO/PVP-SPTO-5.0	CEM	168	75	R.T.	Pt	Pt	[9]
PVA/PEO/PVP-SPTO-2.5	CEM	155	66	R.T.	Pt	Pt	[9]
Nafion^®^117	CEM	183	81	R.T.	Pt	Pt	[9]
Nafion^®^NRE-212	CEM	50	390	80	Au	Au	[10]
Co-PVA-AER	AEM	-	327	60	(Co(OH)_2_-PPy-BP)	(Co(OH)_2_-PPy-BP)	[11]
bipolar junction	BPM	175	630	70	Pd/C+Ni	Pt/C	[12]
pH-gradient-enabled microscale bipolar interface (PMBI)	BPM	175	446	70	NiED/eNF	Pt/C	[13]
layered combination of CEM and AEM bound together	BPM	-	50	30	Au	Pt	[14]

## Data Availability

The data presented in this study are available from the corresponding author upon request.
